# High throughput screening of mesenchymal stem cell lines using deep learning

**DOI:** 10.1038/s41598-022-21653-y

**Published:** 2022-10-20

**Authors:** Gyuwon Kim, Jung Ho Jeon, Keonhyeok Park, Sung Won Kim, Do Hyun Kim, Seungchul Lee

**Affiliations:** 1 Department of Mechanical Engineering, Pohang University of Science and Technology (POSTECH), Pohang, 37673 Republic of Korea; 2 Department of Otolaryngology-Head and Neck Surgery, Seoul St. Mary’s Hospital, College of Medicine, The Catholic University of Korea, 222 Banpo-Daero, Seocho-Gu, Seoul, 06591 Republic of Korea; 3 Department of Biomedicine and Health Science, College of Medicine, The Catholic University of Korea, 222 Banpo-Daero, Seocho-Gu, Seoul, 06591 Republic of Korea; 4 Graduate School of Artificial Intelligence, Pohang University of Science and Technology (POSTECH), Pohang, 37673 Republic of Korea

**Keywords:** Biotechnology, Stem cells, Engineering

## Abstract

Mesenchymal stem cells (MSCs) are increasingly used as regenerative therapies for patients in the preclinical and clinical phases of various diseases. However, the main limitations of such therapies include functional heterogeneity and the lack of appropriate quality control (QC) methods for functional screening of MSC lines; thus, clinical outcomes are inconsistent. Recently, machine learning (ML)-based methods, in conjunction with single-cell morphological profiling, have been proposed as alternatives to conventional in vitro/vivo assays that evaluate MSC functions. Such methods perform in silico analyses of MSC functions by training ML algorithms to find highly nonlinear connections between MSC functions and morphology. Although such approaches are promising, they are limited in that extensive, high-content single-cell imaging is required; moreover, manually identified morphological features cannot be generalized to other experimental settings. To address these limitations, we propose an end-to-end deep learning (DL) framework for functional screening of MSC lines using live-cell microscopic images of MSC populations. We quantitatively evaluate various convolutional neural network (CNN) models and demonstrate that our method accurately classifies in vitro MSC lines to high/low multilineage differentiating stress-enduring (MUSE) cells markers from multiple donors. A total of 6,120 cell images were obtained from 8 MSC lines, and they were classified into two groups according to MUSE cell markers analyzed by immunofluorescence staining and FACS. The optimized DenseNet121 model showed area under the curve (AUC) 0.975, accuracy 0.922, F1 0.922, sensitivity 0.905, specificity 0.942, positive predictive value 0.940, and negative predictive value 0.908. Therefore, our DL-based framework is a convenient high-throughput method that could serve as an effective QC strategy in future clinical biomanufacturing processes.

## Introduction

Recent advances in regenerative medicine and tissue engineering have enabled stem cell therapies to progress to the preclinical and clinical phases in treating various degenerative diseases^1,2^. In particular, mesenchymal stem cells (MSCs) have received considerable attention in tissue regeneration and the treatment of immune system-mediated diseases^3^. Although MSCs exhibit regenerative and immunomodulatory potentials, clinical trials have reported inconsistent therapeutic efficacies^4,5^, likely attributable to functional heterogeneities among MSCs from different donors, variations in MSC production, treatment methods, and recipient conditions; all of these factors exhibit considerable variability^4,6^. In addition, vast numbers of functional stem cells are required for tissue regeneration, in which mass production (using automated cell culture systems) is already commercially available^7,8^. Therefore, effective quality control (QC) of MSC functions is needed for consistent, high-quality, large-scale biomanufacturing of MSCs and their products, as well as successful clinical translation^9,10^. However, current assessment methods lack clinical relevance, adequate assay throughput, and robustness; improved techniques are necessary to better characterize MSC functions.

Recently, several studies have found connections between MSC functions and cell morphology by exploiting advances in high-content microscopic imaging^11^. For example, MSC morphology has been correlated with differentiation capacity^12,13^, motility^14^, and passage number^15,16^. Another field of study seeks to predict in vitro MSC functions via morphological profiling that employs machine learning (ML) algorithms, of which deep learning (DL) algorithms are optimal. Such algorithms have been used to predict MSC osteogenic potential^17,18^, immunosuppressive capacity^19,20^, and microenvironmental interactions^21,22^. These works yield in silico predictions based solely on cell morphology, potentially eliminating the need for costly in vitro/vivo experiments. Candidate therapeutic stem cells can be then identified by selecting lines that are expected to be highly effective and then confirming such efficacy. If efficacy can be predicted using light microscope images periodically acquired during cell culture, the required candidates can be derived without compromising the culture flow.

The efficacy indicators of human mesenchymal tissues may vary depending on the disease that requires therapy. The multilineage differentiating stress-enduring (MUSE) marker evaluates stem cell capacity. MUSE cells are nontumorigenic pluripotent stem cells of human mesenchymal tissues expressing CD-105 and SSEA-3^23^. MUSE cells readily home to damaged tissues and spontaneously differentiate into cells of such tissues, thus repairing the tissues and restoring function^24,25^. Furthermore, MUSE cells exhibit immunomodulatory properties^26^, and DNA repair capacity^24^; these cells have been used to treat cardiovascular and neurological diseases^27,28^. Therefore, it could be considered as an overall marker for evaluating the efficacy of stem cells.

In this study, human neural crest-derived nasal turbinate stem cells (hNTSCs) was used as the cell source for the analysis. In a previous study, after isolation of hNTSCs from inferior turbinate nasal tissue, MSC characteristics were investigated according to the statement of position of the International Society for Cell Therapy^29^. Surface epitope analysis revealed that hNTSCs were negative for CD14, CD19, CD34 and HLA-DR and positive for CD29, CD73 and CD90, indicating a characteristic phenotype of MSCs. The plasticity of hNTSCs has been confirmed in cartilage, bone, adipose and neuronal differentiation conditions^29–33^. hNTSCs have several adventages: donor morbidity is low, and the collection time is short (5–10 min); the cells are derived from the easily acquired turbinate mucosa of patients undergoing surgical treatment of chronic hypertrophic rhinitis (a common condition). The cell numbers are high; these cells exhibit good proliferation, multilineage differentiation potential, and immunomodulatory properties^29,34,35^.

Therefore, we investigated whether high- and low-functioning hNTSC cell lines represented by MUSE markers could be distinguished from DLs through a simple live-cell microscopy imaging process.

## Materials and methods

This section describes cell culture, in vitro assessments, and imaging methods and provides general explanations of DL-based methodologies and evaluation metrics.

### Cell culture/expansion

We used human neural crest-derived nasal turbinate stem cells (hNTSCs); the work was approved by the Ethics Committee (approval no. KC08TISS0341) of Seoul St. Mary’s Hospital of the Catholic University of Korea and all methods were performed in accordance with the relevant guidelines and regulations. Prior to surgery, participants provided written informed consent. hNTSCs were isolated from the turbinate tissue of a human who had undergone partial turbinate resection. The lower turbinate tissue was washed with saline and phosphate-buffered saline (Thermo Fisher Scientific, Waltham, MA, USA) containing an antibiotic-antibacterial solution (Thermo Fisher Scientific), then cut into 1-mm^3^ pieces. Subsequently, the dish was covered with a sterile glass slide. The growth medium was α-minimum essential medium (α-MEM, Thermo Fisher Scientific) with 1% (v/v) penicillin/streptomycin (Invitrogen) and 10% (v/v) fetal bovine serum (Thermo Fisher Scientific). Incubation was performed at 37 °C in a humidified atmosphere under 5% (v/v) CO_2_. The medium was changed at 2–3-day intervals during the 3-week culture period. Finally, the glass cover slide was removed, and cells attached to the culture plate were harvested into a 0.25% (w/v) trypsin solution in 1 mM ethylenediaminetetraacetic acid. hNTSCs were expanded for use in experiments. To analyze MUSE marker expression and deep learning model training in the culture (passage 6), 1 × 10^6^ cells were seeded in 6-well culture plate and a training image was taken. 8 MSC lines were classified into two groups according to MUSE cell markers analyzed by immunofluorescence staining and FACS.

### Flow cytometry

Single cell suspensions were prepared form hNTSCs. Cells were incubated for 30 min at 4 °C with stage-specific embryonicantigen-3 (SSEA-3) antibody (1:100, Abcam, Cambridge, UK, ab16286) followed by Alexa Fluor 633 anti-rat antibody (1:1000, Thermo Fisher scientific, A21094). After incubation with the SSEA-3 antibody, the cells were incubated with CD105 antibody (1:100, PE-conjugated, BD Pharmingen; catalog no. 560839) for 30 min at 4 °C for double staining. The cells were re-suspended in DPBS (Gibco) and acquired through FACS Canto II (BD biosciences) with DIVA software.

### Imaging and preprocessing

Live-cell microscopic imaging was performed using the Lionheart LX automated microscope (BioTek, Winooski, VT, USA) at 37 °C under 5% (v/v) CO_2_. Images were acquired using phase objectives (40× and 100×). Data were acquired as 904 × 1224 8-bit grayscale images, which were preprocessed in terms of hue (H), saturation (S), and value (V) to reduce experimental variations. Images with mean V values > 230 were excluded because their excessive brightness impeded the distinction of cell morphology from the background. The remaining images were adjusted to achieve a mean V value of 130; the images were resized to 226 × 306 pixels using an interpolation method within Python OpenCV Toolbox software. Subsequently, the pixel values were normalized to [0, 1].

### Immunofluorescence staining

The expression levels of SSEA-3 (derived using anti-SSEA-3; 1:300, Abcam, Cambridge, UK, ab16286) and CD-105 (derived using a PE mouse anti-human CD-105; 1:300, BD Pharmingen; catalog no. 560839) were determined via immunofluorescence staining. After 2 days of culture in the medium described above, hNTSCs were fixed in 2% (w/v) paraformaldehyde and washed with phosphate-buffered saline. The cells were then permeabilized with 0.3% (v/v) Triton X-100 (Sigma-Aldrich) and washed with phosphate-buffered saline. After cells had been blocked with 1% (v/v) normal goat serum (Jackson ImmunoResearch Laboratories Inc., West Grove, PA, USA), they were incubated with the primary antibodies mentioned above; they were then incubated with a goat anti-rat Alexa-Fluor 488 antibody (1:1000; Molecular Probes). The nuclei were labeled with 4′,6-diamidino-2-phenylindole (DAPI; Sigma-Aldrich), and fluorescence was observed under a Zeiss LSM510 confocal microscope (Carl Zeiss).

### Deep learning

We compared various convolutional neural network (CNN) models when selecting the appropriate architecture. Specifically, we reviewed VGG19^36^, ResNet50V2^37^, DenseNet121^38^, InceptionV3^39^, and Xception^40^ (Supplementary Fig. 1). The final feature extraction layers of all networks were subjected to single-element averaging using global average pooling^41^, followed by the designation of surrogate classification layers as described in Supplementary Table 1. We leveraged various strategies to train our networks and optimize the trainable parameters. First, we employed transfer learning^42^, which effectively transfers knowledge among different domains and has been successfully employed in several biomedical imaging applications^43^. Our transfer learning strategy featured three steps (Supplementary Fig. 2). In the first step, we obtained pre-trained networks on the ImageNet dataset^44^, in which the feature extraction layers are pre-trained to identify low-level image features (Supplementary Fig. 2a). The trainable parameters of the feature extraction layers were frozen, and a new classification layer (trained using our cell image dataset) classified cell populations into high/low MUSE cells markers using the features learned in the previous step (Supplementary Fig. 2b). Finally, the entire network was fine-tuned at a reduced learning rate (Supplementary Fig. 2c). During training, trainable parameters were initialized using the normal He normalization method^45^ and optimized using the Adam optimizer^46^. Early stopping, L2 regularization^47^, and dropout^48^ were used to avoid overfitting. We then optimized each model’s hyper-parameters using Bayesian optimization (BO)^49^ (Supplementary Fig. 3). Unlike systematic approaches such as the grid and random searches (Supplementary Fig. 3a,b respectively)^50^, BO reduces the computational cost by considering all prior knowledge that facilitates the optimization process (Supplementary Fig. 3c). BO features simultaneous interactions between a surrogate model and an acquisition function. The surrogate model probabilistically models an unknown objective function that maps a set of hyperparameters to the evaluation results by considering previously evaluated samples. The acquisition function suggests a new set of hyperparameters based on exploration of (and exploitation within) the established surrogate model. A Gaussian process^51^ served as the surrogate model, while the expected improvement (EI) algorithm^52^ served as the acquisition function. Further details regarding model training are provided in Supplementary Table 1.

### Evaluation metrics

To evaluate the results of our CNN models, we considered areas under the curves (AUCs), F1 scores, accuracies, sensitivities, specificities, positive predictive values (PPVs), and negative predictive values (NPVs) (Supplementary Table 2).

### Statistical analysis

Statistical difference between two groups were assessed with the unpaired t test (two tailed). All results are presented as the mean ± SD. A p-value of less than 0.05 was considered statistically significant.

## Results

**Figure 1:**
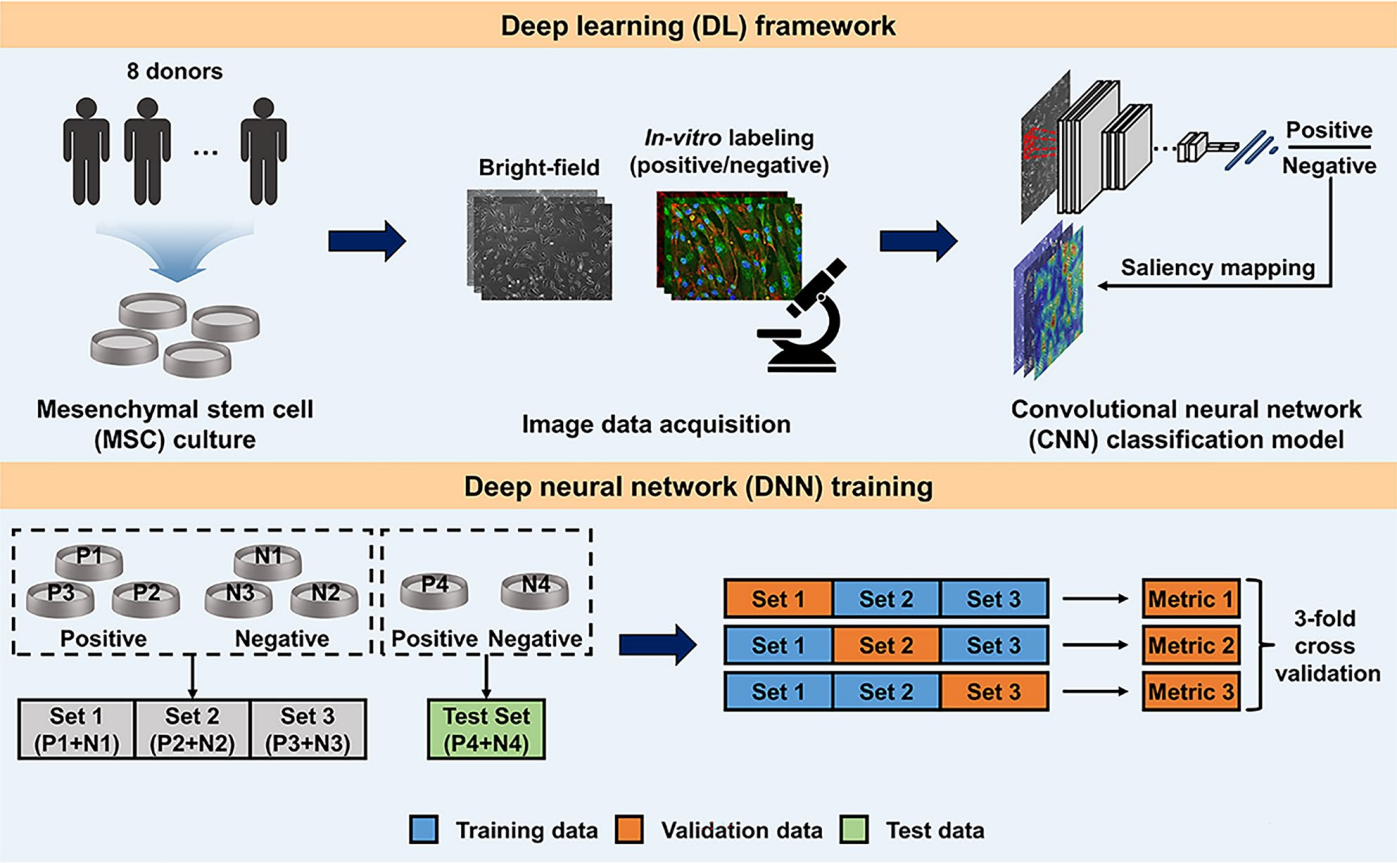
Schematic of the deep learning (DL) framework used to screen for functional mesenchymal stem cell (MSC) lines. Mesenchymal stem cell (MSC) cultures were obtained from eight cell lines from different donors for image data acquisition. To compare the classification performances of various convolutional neural network (CNN) models, threefold cross-validation was conducted by splitting the dataset into non-overlapping subsets.

This section presents and discusses the experimental results of our DL-based MSC screening method. Previous studies found that the number of functional subpopulations was correlated with the overall therapeutic functionality of an MSC batch^53–55^; moreover, subpopulations exhibited distinct morphological features^20,56–58^. We thus hypothesized that microscopic images from functional cell lines contain a higher ratio of such subpopulation distributions; we also hypothesized that a CNN could classify the images based on the morphological characteristics. Therefore, all images were labeled as a whole; images of cell lines with high MUSE cell markers were positive, whereas images of cell lines with low MUSE cell markers were negative. A schematic is shown in Fig. 1. We first conducted multivariate in vitro assessments of eight cell lines from different donors. group with a high expression of MUSE cells (n = 4) and group with a low expression of MUSE cells (n = 4) were identified (Supplementary Fig. 4). Then (as shown in Fig. 1), the total dataset was split into training, validation, and test sets. Three sets of MSC images were obtained for training from six cell lines; each set contained 1,530 images from a positive cell line and 1530 images from a negative cell line. This dataset was used to conduct threefold cross-validation that quantitatively compared the classification performances of various CNN models by splitting the dataset into non-overlapping subsets. We identified the most promising model, then used this model to conduct a further quantitative and qualitative evaluation of an independent dataset with 6,120 cell images/class from test data.

### Characteristics of the classified hNTSC group

Confocal microscopy images confirmed the difference in the MUSE cell markers SSEA-3 and CD-105 between the two groups (Supplementary Fig. 5). Supplementary Fig. 6 showed flow cytometry results of MUSE cells in the six passage showing of SSEA-3, CD105 expression. In FACS analyses, which were performed to confirm this result quantitatively, the results of comparing the MUSE cell markers of the four high groups and the four low groups showed that the MUSE cell marker expression was approximately twofold higher in the high group (3.5%) than in the low group (1.7%). It was confirmed that there was a significant difference between the forces (Supplementary Fig. 6b).

### Bayesian optimization and model comparison

**Figure 2:**
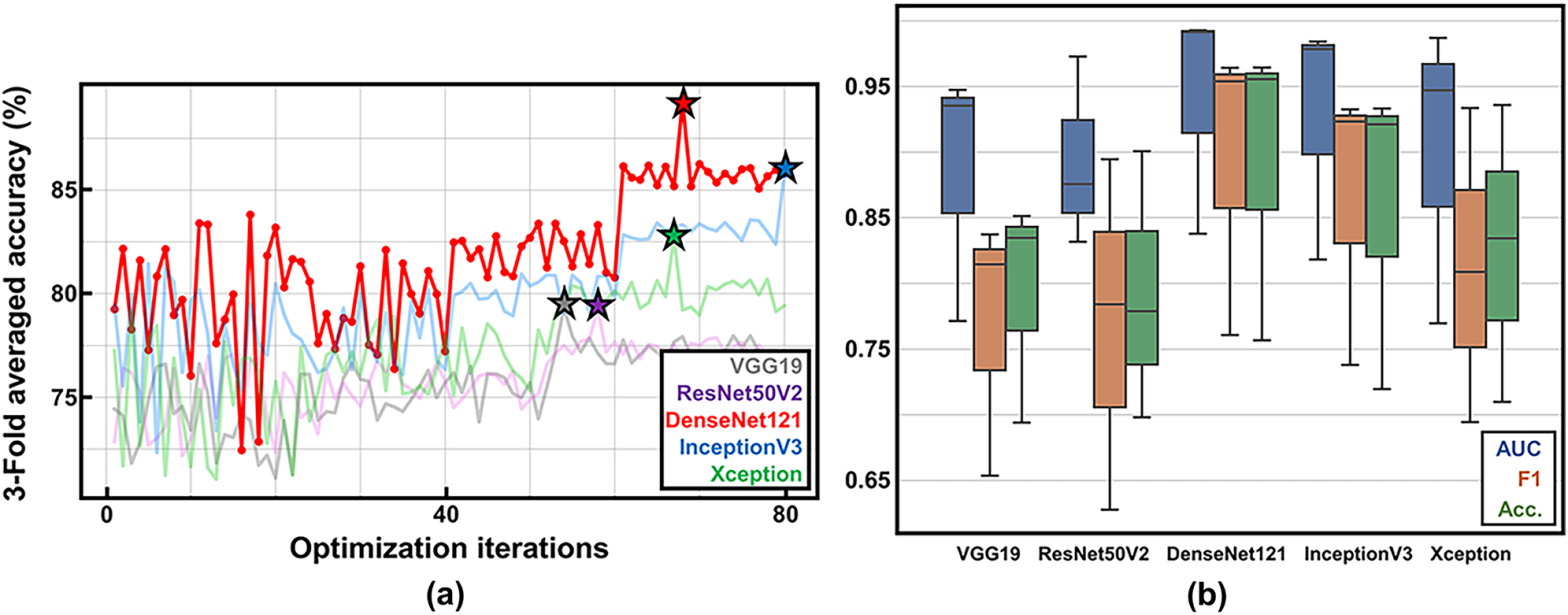
Hyperparameter optimization of comparative convolutional neural network (CNN) models during 80 Bayesian optimization (BO) iterations revealed by mean threefold cross-validation accuracies. (**a**) A progressive plot illustrating the BO process (stars indicate points of optimal performance and DenseNet121 was considered the best model) and (**b**) the threefold cross-validation metrics for each model after BO. AUC, the area under the curve; and Acc., accuracy.

Using the search spaces of the hyperparameters listed in Supplementary Table 1, we subjected various models to BO (we recorded the mean threefold cross-validation accuracies); the results are shown in Fig. 2 and Table 1 while the confusion matrices are shown in Supplementary Fig. 7. BO utilizes prior knowledge concerning the optimization process, then searches for new hyperparameter sets (Fig. 2a). The threefold cross-validation accuracy gradually increased with increasing optimization; early, accuracy was low, and variance was high; later, accuracy was high and appeared to converge for all models. The optimal hyperparameter sets are starred; the values are listed in Supplementary Table 1. As shown in Fig. 2b, DenseNet121 outperformed the other methods in most evaluation metrics. When analyzed with the DenseNet121 model, AUC 0.941 ± 0.089, F1 0.893 ± 0.115, accuracy 0.892 ± 0.117, sensitivity 0.885 ± 0.099, specificity 0.899 ± 0.138, PPV 0.902 ± 0.133, and NPV 0.884 ± 0.104 were obtained. Comparing the best-performing CNN with others, DenseNet121 had an AUC average score of 0.941, while VGG19 had an AUC score of 0.885; VGG19 exhibited the worst performance, reflecting the consensus that deeper networks benefit from their ability to model mappings of higher complexity^36^. InceptionV3 and Xception models performed poorly compared to DenseNet121; these findings suggested that their multi-scale convolution operations overexpressed our cell image dataset, thus leading to overfitting. Although VGG19 exhibits the fastest inference speed due to its relatively simple architecture [964 frames per second (FPS)], all models perform above 300 FPS, which is sufficient for real-time implementations. The threefold cross-validation results indicate that our method robustly managed the heterogeneities of cell lines. Below, we focus exclusively on DenseNet121.

**Table 1:** Threefold cross-validation metrics of all convolutional neural networks (CNNs) after Bayesian optimization (BO).

	AUC	F1	Accuracy	Sensitivity	Specificity	PPV	NPV	FPS
VGG19	0.885 ± 0.098	0.768 ± 0.1	0.793 ± 0.086	0.689 ± 0.099	0.897 ± 0.075	0.869 ± 0.01	0.744 ± 0.076	**964 ± 48**
ResNet50V2	0.893 ± 0.072	0.769 ± 0.134	0.792 ± 0.102	0.718 ± 0.182	0.867 ± 0.103	0.846 ± 0.097	0.765 ± 0.11	458 ± 61
DenseNet121	**0.941 ± 0.089**	**0.893 ± 0.115**	**0.892 ± 0.117**	0.885 ± 0.099	0.899 ± 0.138	**0.902 ± 0.133**	**0.884 ± 0.104**	378 ± 25
InceptionV3	0.927 ± 0.094	0.865 ± 0.11	0.858 ± 0.12	**0.886 ± 0.084**	0.83 ± 0.159	0.846 ± 0.135	0.874 ± 0.103	519 ± 30
Xception	0.901 ± 0.116	0.812 ± 0.12	0.827 ± 0.113	0.754 ± 0.13	**0.9 ± 0.121**	0.886 ± 0.133	0.787 ± 0.11	364 ± 34

AUC, the area under the curve; PPV, positive predictive value; NPV, negative predictive value; FPS, frames per second.

Significant values are given in bold.

### Independent test set evaluation

**Figure 3:**
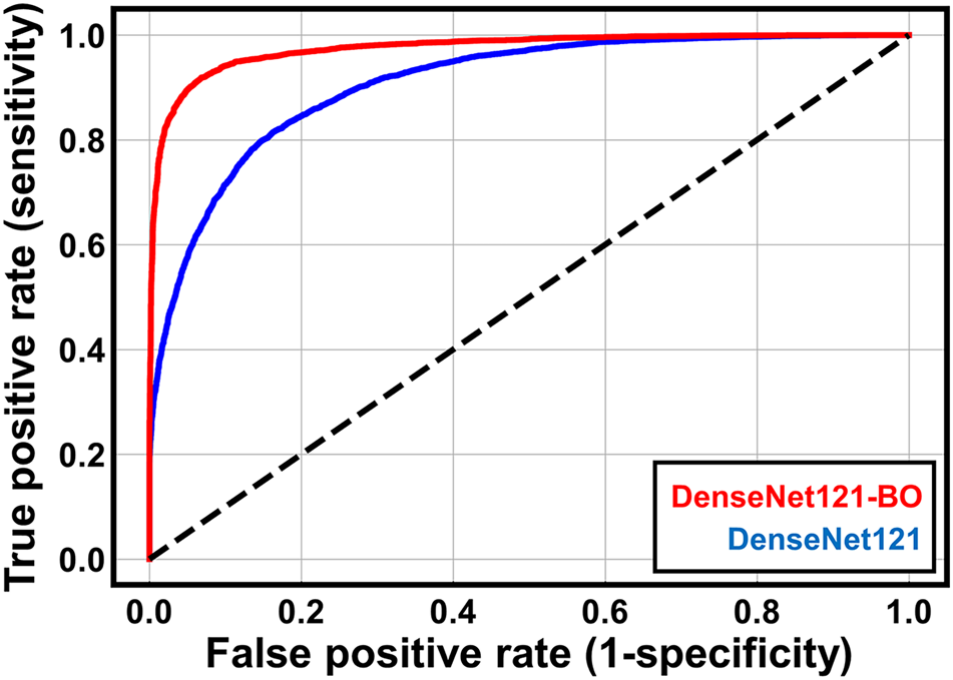
Receiver operating characteristic (ROC) curves of DenseNet121 operating on the independent test dataset before and after Bayesian optimization (BO). The prediction performance of DenseNet121 was improved after the BO process.

**Figure 4:**
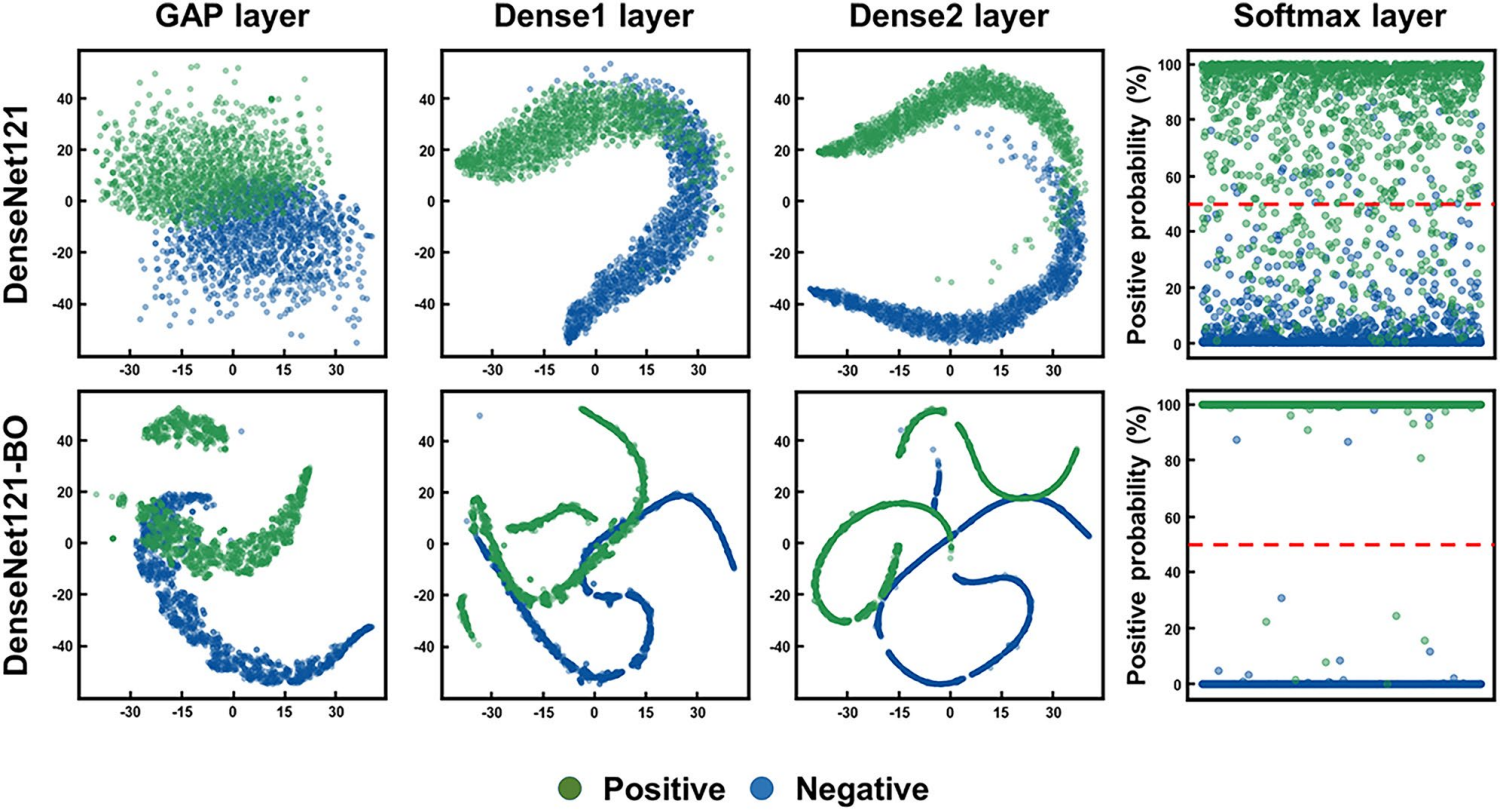
t-stochastic neighborhood embedding (t-SNE) visualizations of the feature outputs of different classification layers and the distributions of softmax outputs of the final classification layers before and after Bayesian optimization (BO). As the layers progressed within the models and after the BO process, the distributions of the two classes gradually became disentangled. GAP, global average pooling.

**Figure 5:**
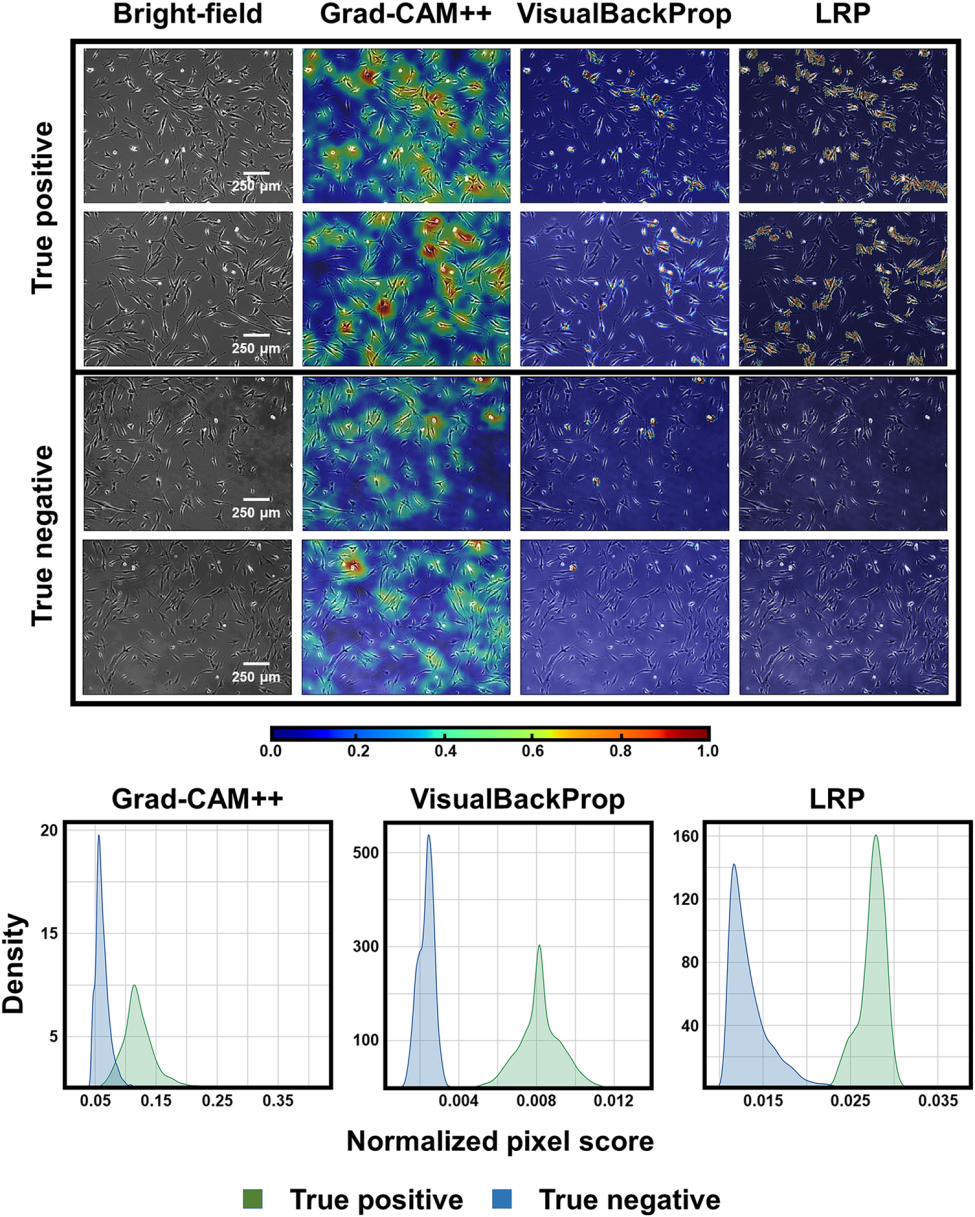
Interpretation of convolutional neural network (CNN) analyses using three saliency mapping algorithms: gradient-based class activation mapping (Grad-CAM++), visual back-propagation (VisualBackProp), and layer-wise relevance propagation (LRP). The Grad-CAM++ exhibited overlaps between the two classes due to the algorithms’ propensity to highlight large ROIs, while VisualBackProp and LRP provided distinct distributions. Normalized pixel score distributions are shown for each algorithm.

All images from the threefold dataset were merged and used to retrain DenseNet121 with the hyperparameter set suggested by BO. We evaluated the performances of the optimized DenseNet121 (termed DenseNet121-BO) and the pre-optimized DenseNet121 (trained using the initial hyperparameters of Supplementary Table 1) using an independent test dataset derived from the remaining cell lines (Fig. 3 and Table 2; the confusion matrices are shown in Supplementary Fig. 8). Quantitatively, all metrics (including receiver operating characteristic curves) improved after BO. The optimized DenseNet121 model was improved to AUC 0.975, accuracy 0.922, F1 0.922, sensitivity 0.905, specificity 09.42, PPV 0.940, and NPV 09.08. The FPS deteriorates slightly since the number of dense neurons in the classification layers increases after BO (Supplementary Table 1). However, FPS values above 300 are sufficient for real-time screening. The prediction performance of DenseNet121 after BO process would be expressed with an AUC score of 0.975. To further analyze the effects of BO, we examined the feature representations of all classification layers before and after BO using the t-stochastic neighborhood embedding algorithm^59^. Figure 4 shows the embedded feature distributions of the test dataset in the global average pooling and dense layers of our model; the distributions of the two classes gradually became disentangled as they progressed within the layers. Notably, the layer distributions became more distinct after BO because BO optimizes the dense layer structures (Supplementary Table 1). Overall, BO improved the generalization afforded by our DenseNet121 model. Finally, we used several saliency mapping algorithms to highlight regions of interest (ROIs) that substantially contributed to the classification results. For this purpose, we used gradient-based class activation mapping (Grad-CAM++)^60^, layer-wise relevance propagation (LRP)^61^, and visual back-propagation (VisualBackProp)^62^. Sample true-positive (TP) and true-negative (TN) images (with ROIs) are shown in Fig. 5. All three algorithms highlighted similar ROIs within the images; the TP images featured large ROIs (upper two rows), and the TN images featured small ROIs (bottom two rows). We calculated the normalized pixel scores of all images via saliency mapping by thresholding each pixel value below 0.5 to 0, then averaging the remaining values. The normalized pixel score distributions revealed clear differences between TP and TN images. The Grad-CAM++ distribution illustrates overlaps between the two classes due to the algorithm’s tendency to highlight relatively large ROIs, resulting in large normalized pixel scores. The opposite was true of VisualBackProp and LRP; the ROIs of TP images were small, and the ROIs of TN images were nearly absent, which led to distinct distributions and small normalized pixel scores.

**Table 2:** Quantitative metrics of DenseNet121 derived using an independent test dataset before and after Bayesian optimization (BO).

	AUC	F1	Accuracy	Sensitivity	Specificity	PPV	NPV	FPS
DenseNet121-BO	**0.975**	**0.922**	**0.923**	**0.905**	**0.942**	**0.940**	**0.908**	324
DenseNet121	0.908	0.820	0.825	0.800	0.849	0.842	0.810	**389**

AUC, the area under the curve; PPV, positive predictive value; NPV, negative predictive value; FPS, frames per second.

Significant values are given in bold.

## Discussion

In this study, we divided stem cell lines with different MUSE cell markers into two groups (high vs. low MUSE expression) when establishing our DL model that was trained using simple light microscopic images. Evaluation of hNTSCs with distinct patterns of MUSE cell marker expression enabled the separation of hNTSCs donor variation. It was verified that SSEA-3 and CD105 of the high MUSE expression group were higher than those of the low MUSE expression group by using immunofluorescence staining, and it was confirmed more clearly by FACS analysis. The optimized DenseNet121 DL model showed AUC 0.975, accuracy 0.922, F1 0.922, sensitivity 0.905, specificity 09.42, PPV 0.940, NPV 0.908 results, therefore, it is possible to discriminate groups with high or low expression of MUSE cell markers from cell images with very high accuracy.

Although ML-based methods effectively predict in vitro MSC function, several aspects require investigation; these motivated our work. First, DL-based principles should be used to create an end-to-end framework that optimizes feature extraction^47^. ML algorithms require manual identification of morphological features in single-cell images; thus, high-content imaging followed by extensive image-processing is necessary^63^. This approach limits screening throughput and hinders the complete exploitation of high-dimensional morphological information inherent in the image data. Additionally, manually identified features are experiment-dependent; they are not generalizable to cells produced under different conditions^19^. Second, an optimal method must be generalizable to MSCs from various donors evaluated under different experimental conditions. Although a previous study^64^ successfully utilized CNNs that evaluated cell morphology to predict the immunofluorescence levels of nine different surface markers, the proof-of-concept study reported results from only a single donor culture after only a few passages at a low seeding density. To address the above limitations, we proposed a novel DL-based method to identify MSC lines to high/low MUSE cells markers using live microscopic images. Previous reasearch^20,56–58^ showed that the proportions of morphological subpopulations reflect MSC therapeutic functions. Also, the number of functional subpopulations was correlated with the overall therapeutic functionality of an MSC batch^53–55^. We thus hypothesized that CNNs could be used to examine whole-labeled, live-cell microscopic images of MSC populations, then identify the in vitro characteristics of those populations. By leveraging various DL principles, we circumvented the extensive imaging procedures that were required by previous ML approaches; our MSC screening strategy is much faster performing above 300 FPS. We initially established and optimized several CNN models, using BO; DenseNet121 was considered the best model (Fig. 2, Table 1). Our model demonstrated AUCs > 0.94 during validation involving MSC images from eight different cell lines. We then explored the interpretations of the CNN using various saliency mapping algorithms; we sought to ensure that our method was reliable. When used with single-cell morphological profiling^63^, we provided insights that could serve as potential biomarker candidates for identifying desired subpopulations. The visualization results strongly supported our initial hypothesis: CNNs can classify whole-labeled images into high/low MUSE cells markers by examining the morphological features of subpopulations, as well as the proportions of such subpopulations. Our DL-based method is generalizable and offers high throughput. Identifying surface markers unique to these subpopulations^65^ would enable their enrichment using methods such as fluorescence-activated cell sorting (FACS)^53^. Finally, our approach will facilitate inter-and intra-laboratory research; morphological profiling is more cost-effective and less sensitive to experimental conditions compared with conventional immunohistochemistry- and flow cytometry-based methods^66^.

## Conclusions

We developed a DL-based in silico method to identify MSC lines to high/low MUSE cells markers; this method facilitates high-throughput QC during biomanufacturing. Through this process, function screening, replacing some of the in vivo and in vitro tests performed to confirm the function of each cell stem line can be accomplished. Therefore, our non-invasive, automated in silico system, which uses simple microscopy alone, will aid cell biomanufacturing and translation to cell therapies.

## Supplementary Information

Supplementary Figure 1.

Supplementary Figure 2.

Supplementary Figure 3.

Supplementary Figure 4.

Supplementary Figure 5.

Supplementary Figure 6.

Supplementary Figure 7.

Supplementary Figure 8.

Supplementary Table 1.

Supplementary Table 2.

## Data Availability

The datasets used and/or analysed during the current study available from the corresponding author on reasonable request.
