# Supplementary figures and images for: High throughput screening of mesenchymal stem cell lines using deep learning
Source: Sci Rep. 2022 Oct 20;12:17507. doi: 10.1038/s41598-022-21653-y (PMC9584889; doi:10.1038/s41598-022-21653-y)

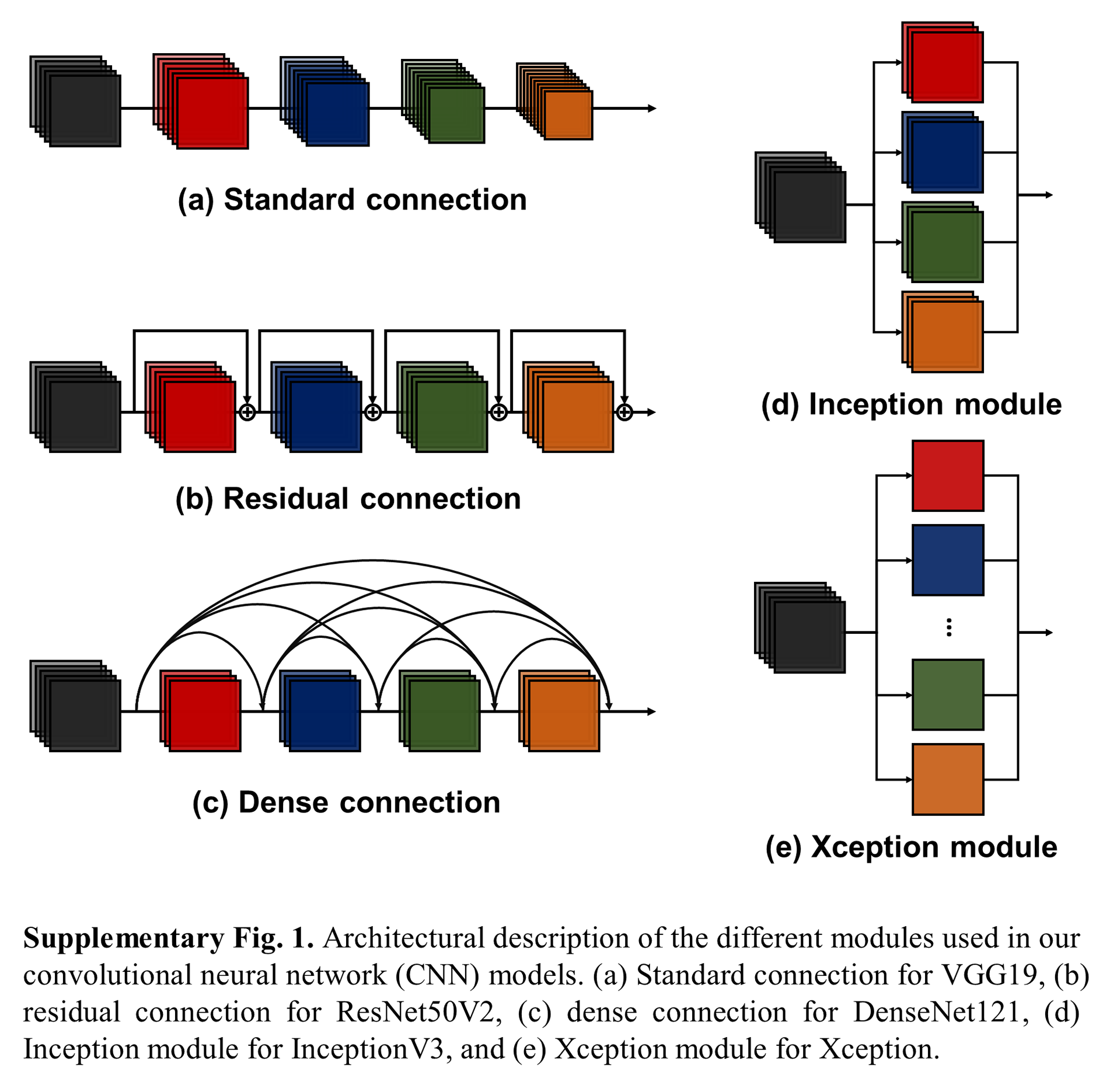

Supplement: Supplementary file 1 — Supplementary Figure 1. [file 41598_2022_21653_MOESM1_ESM.tif]

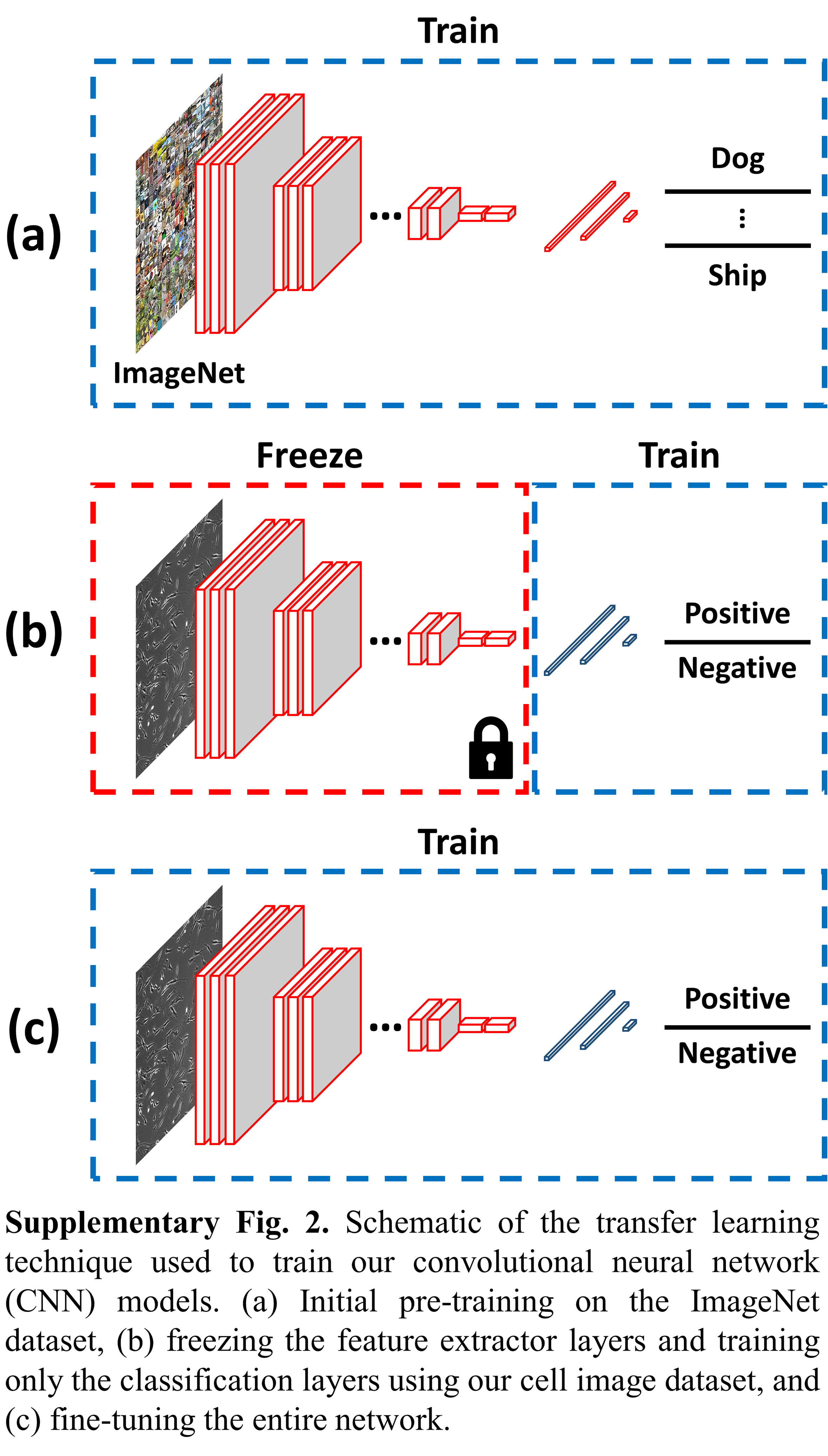

Supplement: Supplementary file 2 — Supplementary Figure 2. [file 41598_2022_21653_MOESM2_ESM.tif]

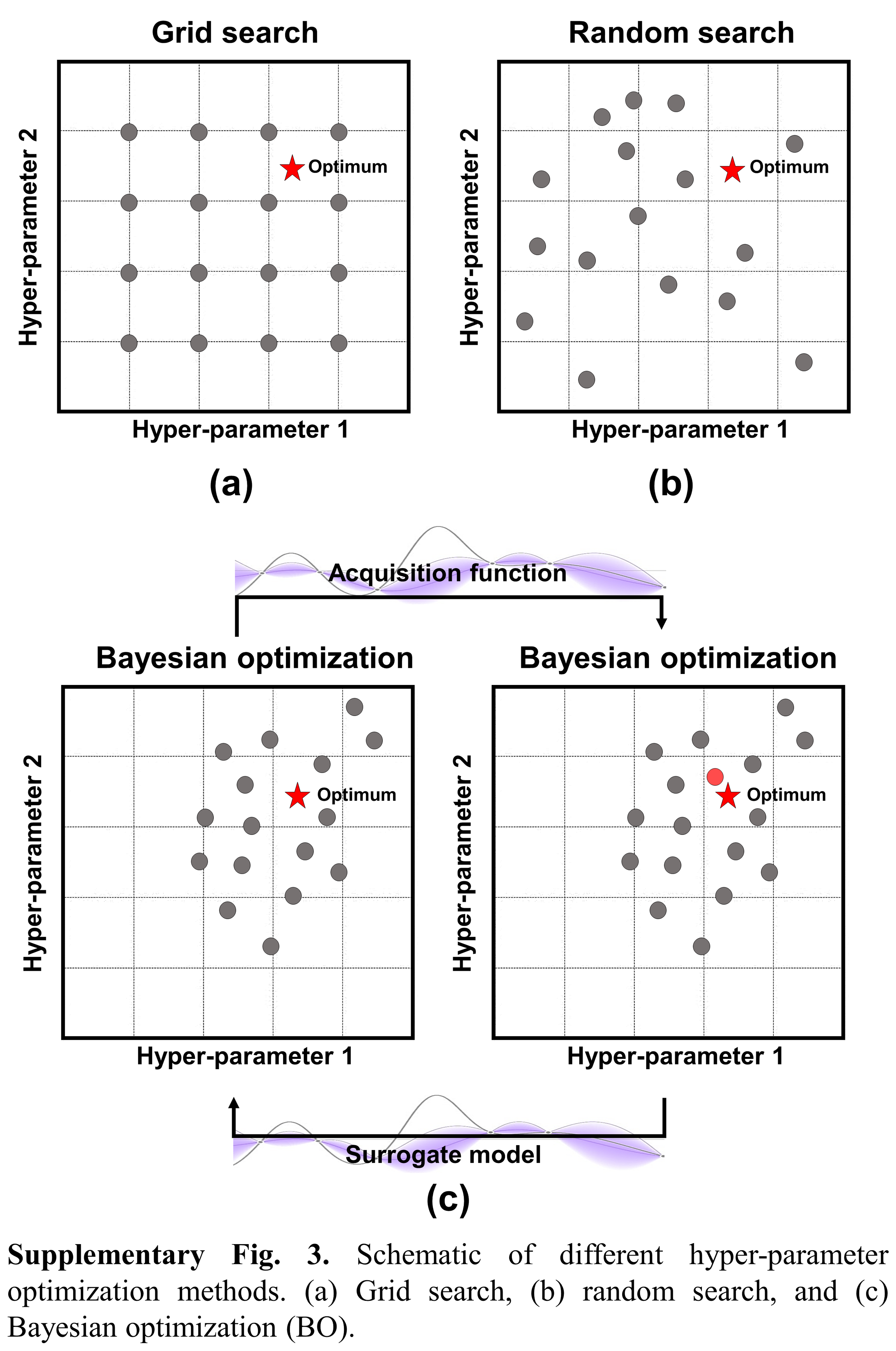

Supplement: Supplementary file 3 — Supplementary Figure 3. [file 41598_2022_21653_MOESM3_ESM.tif]

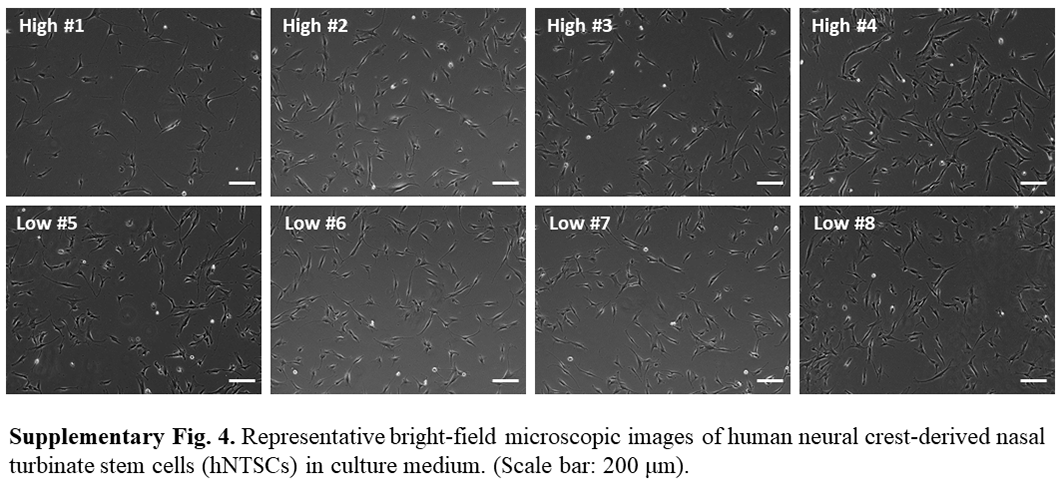

Supplement: Supplementary file 4 — Supplementary Figure 4. [file 41598_2022_21653_MOESM4_ESM.tif]

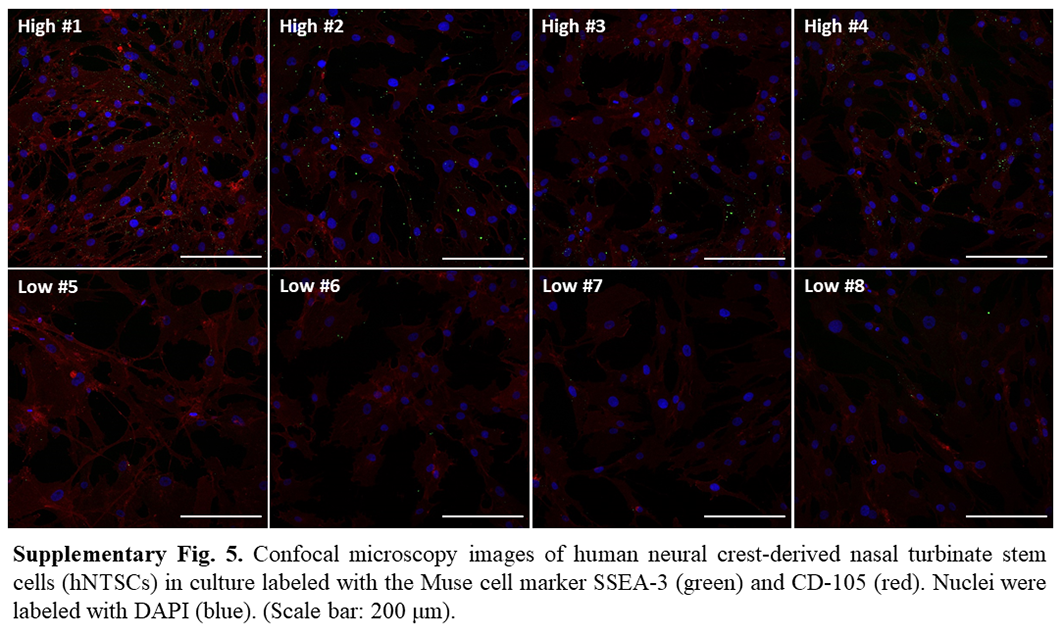

Supplement: Supplementary file 5 — Supplementary Figure 5. [file 41598_2022_21653_MOESM5_ESM.tif]

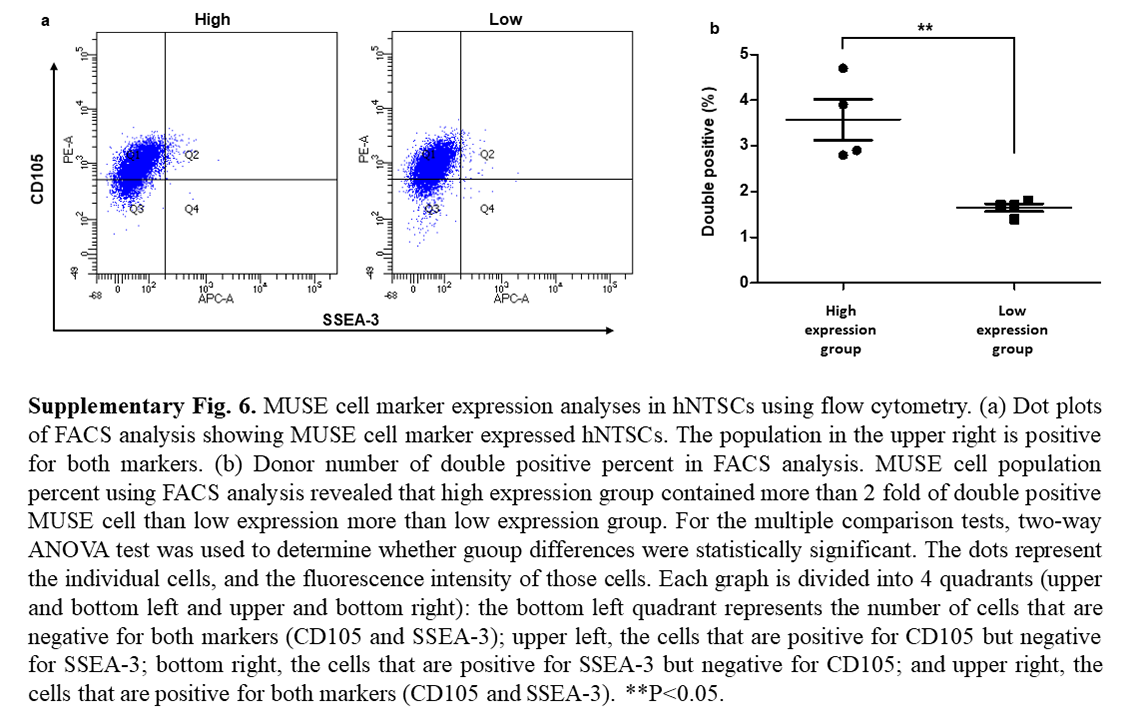

Supplement: Supplementary file 6 — Supplementary Figure 6. [file 41598_2022_21653_MOESM6_ESM.tif]

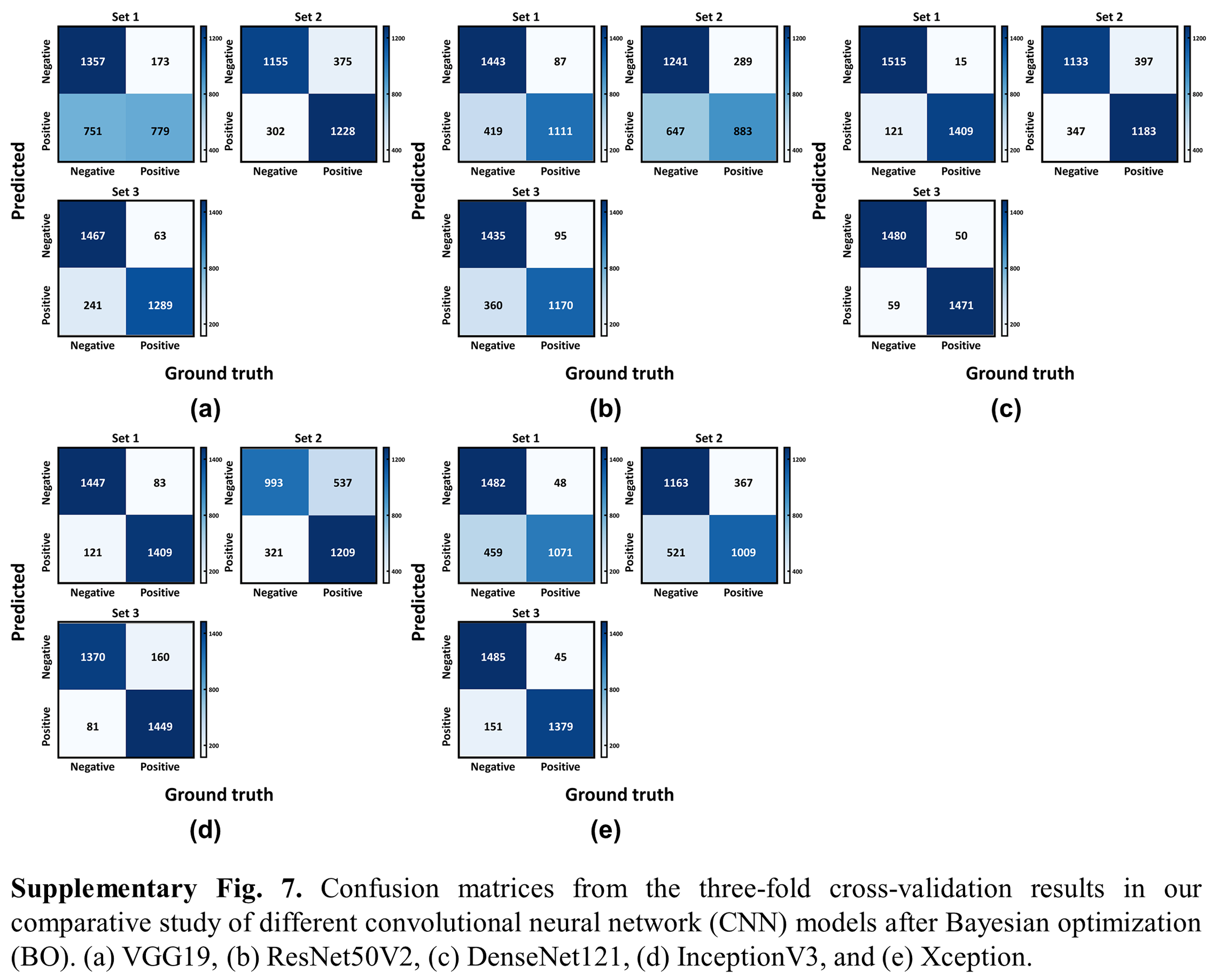

Supplement: Supplementary file 7 — Supplementary Figure 7. [file 41598_2022_21653_MOESM7_ESM.tif]

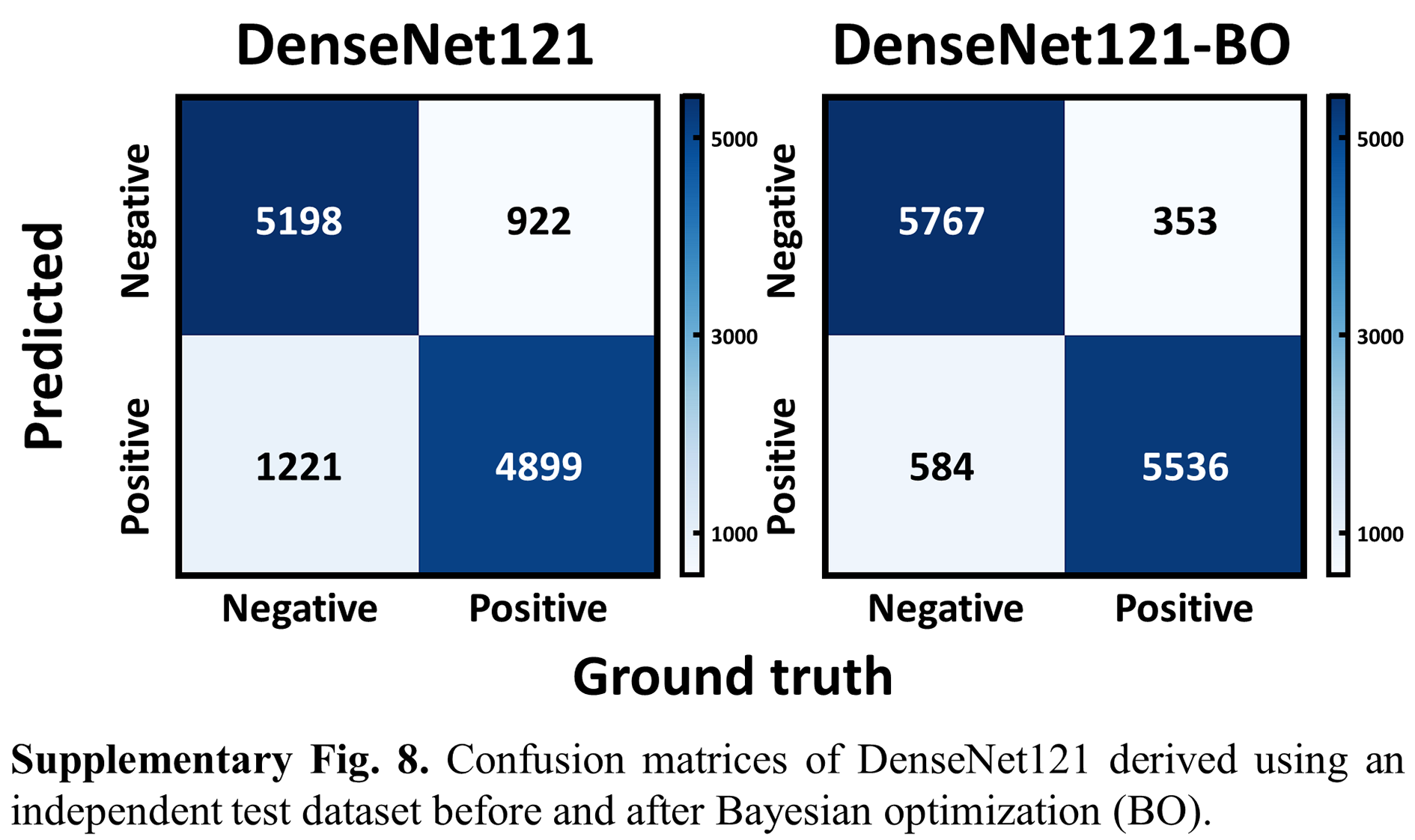

Supplement: Supplementary file 8 — Supplementary Figure 8. [file 41598_2022_21653_MOESM8_ESM.tif]
