# Supplementary material for: High throughput screening of mesenchymal stem cell lines using deep learning
Source: Sci Rep. 2022 Oct 20;12:17507. doi: 10.1038/s41598-022-21653-y (PMC9584889; doi:10.1038/s41598-022-21653-y)
Supplement: Supplementary file 9 — Supplementary Table 1. [file 41598_2022_21653_MOESM9_ESM.docx]

**Supplementary Table 1.** Training details of our convolutional neural network (CNN) models. CNN models were implemented using Python 3.6.8 with a Keras backend. Pre-trained feature extraction layers were obtained from Keras Applications, and structural descriptions can be found at https://keras.io/api/applications/. Model training is conducted on an NVIDIA TITAN Xp GPU, and model evaluation is performed on an Intel®CoreTM i5-8400 x64 CPU 16.0GB RAM CPU. GAP, global average pooling.

| Convolutional neural network (CNN) training | | | | | | | | |
| --- | --- | --- | --- | --- | --- | --- | --- | --- |
| Hyper-parameter | Type | Surrogate parameter | Search space | VGG19 | ResNet50V2 | DenseNet121 | InceptionV3 | Xception |
| Batch size | Model training | 32 | [32, 128] | 43 | 112 | 54 | 78 | 39 |
| Initial learning rate | Model training | 5e-4 | [1e-4, 1e-3] | 3.1e-4 | 2.9e-4 | 7.2e-4 | 7.8e-4 | 4.3e-4 |
| Fine-tuning learning rate | Model training | 5e-6 | [1e-6, 1e-5] | 2.9e-6 | 3.8e-6 | 7.3e-6 | 1.9e-6 | 9.7e-6 |
| Weight decay | Regularization | 5e-6 | [1e-6, 1e-5] | 1.8e-6 | 2.3e-6 | 3.4e-6 | 3.2e-6 | 4.7e-6 |
| Dropout rate 1 (GAP) | Regularization | 0.15 | [0.1, 0.5] | 0.34 | 0.27 | 0.12 | 0.33 | 0.41 |
| Dropout rate 2 (Dense) | Regularization | 0.8 | [0.5, 0.9] | 0.59 | 0.72 | 0.78 | 0.88 | 0.63 |
| Dropout rate 3 (Dense) | Regularization | 0.6 | [0.5, 0.9] | 0.54 | 0.71 | 0.66 | 0.76 | 0.68 |
| Dropout rate 4 (Dense) | Regularization | 0.6 | [0.5, 0.9] | 0.64 | 0.59 | 0.72 | 0.78 | 0.53 |
| Dense neuron 1 | Model structure | 256 | [256, 512] | 272 | 312 | 262 | 319 | 411 |
| Dense neuron 2 | Model structure | 128 | [128, 256] | 181 | 132 | 144 | 138 | 174 |
| Dense neuron 3 | Model structure | 2 | - | 2 | 2 | 2 | 2 | 2 |
