# Supplementary material for: High throughput screening of mesenchymal stem cell lines using deep learning
Source: Sci Rep. 2022 Oct 20;12:17507. doi: 10.1038/s41598-022-21653-y (PMC9584889; doi:10.1038/s41598-022-21653-y)
Supplement: Supplementary file 10 — Supplementary Table 2. [file 41598_2022_21653_MOESM10_ESM.docx]

**Supplementary Table 2.** Statistic metrics for evaluating the prediction results of our convolutional neural network (CNN) models. TN, true negative; FN, false negative; FP, false positive; and TP, true positive.

| Evaluation metrics | |
| --- | --- |
| Metric | Description |
| The area under the curve (AUC) | The area under the receiver operator characteristics (ROC) plot. |
| F1 score | $\frac{2\times TP}{(2\times TP+FP+FN)}$ |
| Accuracy | $\frac{(TP+TN)}{(TP+TN+FP+FN)}$ |
| Sensitivity | $\frac{TP}{(TP+FN)}$ |
| Specificity | $\frac{TN}{(TN+FP)}$ |
| Positive predictive value (PPV) | $\frac{TP}{(TP+FP)}$ |
| Negative predictive value (NPV) | $\frac{TN}{(TN+FN)}$ |
